# The CDK1-Related lncRNA and CXCL8 Mediated Immune Resistance in Lung Adenocarcinoma

**DOI:** 10.3390/cells11172688

**Published:** 2022-08-29

**Authors:** Jinmin Xue, Yang Song, Wenwen Xu, Yuxi Zhu

**Affiliations:** 1 Department of Oncology, The First Affiliated Hospital of Chongqing Medical University, Chongqing 400016, China; 2 Department of Oncology, Jinshan Hospital of the First Affiliated Hospital of Chongqing Medical University, Chongqing 400016, China; 3 Chongqing Clinical Cancer Research Center, Chongqing 400016, China

**Keywords:** lung adenocarcinoma, CDK1, LINC00261, CXCL8

## Abstract

Background: Limited therapeutic options are available for advanced LUAD without driver gene mutations. Anti-CDK therapy has shown effectiveness in several kind of cancers, however, the mechanisms still need to be elucidated. Materials and Methods: The lncRNA associated with CDK1 and the immunomodulatory factors that regulate CDK1 were found by bioinformatics analysis and experimental verification. The prognostic model and immune resistance mechanism of lung adenocarcinoma were revealed by single cell analysis, immune infiltration analysis, and signal pathway analysis. Results: LINC00261 was found to be an important CDK1-related lncRNA with a better prognosis in LUAD. In addition, high CDK1 expression indicates a poor immunotherapy response, which may be associated with overexpression of CXCL8. CXCL8 decreased in patients who were immunotherapy-responsive but increased in patients who were immunotherapy-resistant. Signaling pathway analysis suggested that increased CXCL8 and decreased LINC00261 may participate in hypoxia-induced tumor angiogenesis and cause a poor prognosis for the patients. CXCL8 and CDK1 may change G2-M transformation and EMT and promote tumor proliferation. Conclusion: This study explained that LINC00261, CDK1, and CXCL8 may have a mutual regulation relationship, which affects the occurrence of LUAD and the efficacy of immunotherapy.

## 1. Introduction

Lung cancer is the most common and fatal cancer in men [1]. Lung adenocarcinoma (LUAD) is an important component of lung cancer, which is often accompanied by poor prognosis. Targeted therapy and immunotherapy have prolonged survival and improved quality of life for some patients with lung adenocarcinoma [2,3]. However, in clinical practice, patients with a negative drive gene and immune checkpoint inhibitor (ICI) treatment resistance account for a large proportion of patients with lung adenocarcinoma. Novel effective treatments are needed for improvement in the clinical outcomes of these patients.

The core of the cell cycle regulatory system is a group of cyclin-dependent kinases (CDKs) [4], each of which is activated at a specific time in the cell cycle and drives the completion of the cell cycle by phosphorylating the corresponding substrate. They are divided into two categories: (1) cell-cycle-associated CDKs (CDK1, CDK2, CDK4, and CDK6) that directly regulate cell cycle progression; and (2) transcriptionally associated CDKs (CDK7, CDK8, CDK9, CDK12, and CDK13) [5]. CDK4/6 is considered to be the key to initiate G1/S phase progression of the cell cycle, and it is related to the phosphorylation of retinoblastoma (RB) gene and related proteins [6]. This series of actions stimulates E2F, and active E2F induces the expression of cyclin E and other proteins that promote CDK2 activity, further leading to RB phosphorylation [7]. On the other hand, transcription-associated CDKs regulate gene expression by phosphorylating some subunits of RNA polymerase II [5]. For example, CDK12 can phosphorylate serine residues within the C-terminal domain of RNA polymerase II and directly regulate transcription. CDK12 inactivation defines a special class of castration-resistant prostate cancer, and it is thought to benefit from immune checkpoint immunotherapy [8].

CDK-inhibitors have shown significant anti-tumor activity in basic research, among which CDK4/6 inhibitors have been clinically approved for breast cancer treatment [9]. However, the development of other CDK inhibitors has been troubled, possibly because of some unrecognized mechanism.

During the cell cycle, there are two checkpoints that prevent cells from repairing damaged DNA to maintain genome integrity. The ATM-CHK2-p53 pathway controls the G1 checkpoints, and the ATR-CHK1-Wee1 pathway controls the S and G2/M checkpoints [10]. Many cancer cells have defective G1 checkpoints and are, therefore, much more dependent on the G2 checkpoints than normal cells. The complex of CDK1 and cyclin B is involved in the key link of G2-M phase transition [11], so the in-depth study of CDK1 is particularly necessary. Preclinical studies have found that anti-CDK1 treatment can promote the antitumor response of sorafenib in hepatocellular carcinoma [12]. However, the relevant research targeting CDK1 is mainly focused on multi-targeted inhibitors against the CDK family. A large number of adverse reactions were found, and the effective rate of CDKs inhibitors was very different from the theory. Exploring the specific signaling pathway of CDK1 and developing targeted drugs is a potential anti-tumor drug research direction.

Current studies generally believe that lncRNA regulatory networks are involved in the development in cancers [13] and immune resistance. We tried to search for potential lncRNAs and immune regulators associated with CDK1, by means of bioinformatics analysis and clinical data analysis, to further explore the new phenotypic characteristics of LUAD and provide potential ideas for CDK1-related translational research.

## 2. Materials and Methods

### 2.1. Data Source and Processing

A part of the clinical information and gene-sequencing data involved in this study were obtained from TCGA database (https://portal.gdc.cancer.gov/, accessed on 17 March 2022). Another part of clinical specimens and hematological specimens were collected from lung adenocarcinoma patients who fulfilled the research conditions at the Department of Oncology, the First Affiliated Hospital of Chongqing Medical University. All patient medical records and biospecimens were obtained during the previous treatment process, which did not adversely affect the patients’ rights or health, and the study was approved by the ethics of the investigator’s unit. Informed consent was waived for non-interventional retrospective study by the Ethics Committee of the First Affiliated Hospital of Chongqing Medical University. Ethical approval number is 2021-347. The Human Protein Atlas database (https://www.proteinatlas.org/, accessed on 17 March 2022) [14] strives to provide tissue and cellular distribution information for various classes of human proteins. We explored some additional information on the target genes with this database.

### 2.2. Exploration of CDK1-Associated lncRNAs

#### 2.2.1. Exploration of CDK1-Associated Differentially Expressed Genes (DEGs)

RNAseq data of lung adenocarcinoma patients in TCGA were obtained with R software, and single gene differential expression analysis about CDK1 was performed with DEseq2 package of R [15]. The differential expression profiles of 56,493 DEGs were obtained. These included 1448 differentially expressed miRNAs as well as 14,077 differentially expressed lncRNAs. Statistically significant differentially expressed RNAs were further selected (set as follows: log_2_ foldchange > 1 or log_2_ foldchange < −1, *p* < 0.05). In addition, volcano plots were depicted with the ggplot2 package [16] for visualization. Next, the top 30 DEmiRNAs and DElncRNAs with the most significant expression differences were individually selected to draw a CDK1-associated co-expression heatmaps.

#### 2.2.2. Dentification of Hub-lncRNA

According to the hypothesis that lncRNA could indirectly regulate mRNA expression by competing with miRNA as a natural sponge in the cytoplasm [17], at ENCORI (http://starbase.sysu.edu.cn/index.php, accessed on 11 April 2022) [18] online platform, we used differentially expressed miRNAs to predict potentially relevant lncRNAs, respectively. Meanwhile, the VennDiagram package [19] in R software was used to compare the predicted genes with the previous lncRNAs of the DEGs, and the overlapped genes were included for the next analysis. In the next step, a lncRNA-miRNA regulatory network was constructed by integrating lncRNA-miRNA pairs. LnCAR (https://lncar.renlab.org/explorer, accessed on 14 May 2022) is a comprehensive database on lncRNAs [20], which was used to perform survival analysis of lncRNAs in the network. In addition, survivable lncRNAs were defined as Hub-lncRNAs.

#### 2.2.3. Validation of RNA Expression Levels and Clinical Relevance

First, we utilized TCGA data to verify the expression profiles of Hub-lncRNA and CDK1 in tumor tissues and normal lung tissues. The normality test results showed that the selected samples did not satisfy the normality test (*p* < 0.05), so the Kruskal–Wallis test was further selected. The Bonferroni method was used to correct the results for multiple hypothesis testing (Dunn’s test) at the significance level. Further subgrouping was performed according to target molecular expression. Chi-squared test and t-test were used to explore the correlation between target molecular expression and clinical stage, gender, age, race, and smoking in lung adenocarcinoma patients (*p* < 0.05 was considered significant). Finally, we collected surgical specimens from patients with lung adenocarcinoma and used RT-qPCR method to validate the expression difference of CDK1 and Hub-lncRNA between lung adenocarcinoma tissues and adjacent noncancerous tissues.

#### 2.2.4. Molecular Correlation Analysis

R software was used to call the data of lung adenocarcinoma patients from TCGA database. Next, the corrplot package was invoked to explore the correlation between the expression quantity of each molecule using spearman’s principle.

### 2.3. CDK1 and the Immune Response

#### 2.3.1. Prediction of Responsiveness to ICIs

We applied the ggplot2 and ggpubr packages to predict immune checkpoint inhibitor responsiveness using the Tumor Immune Dysfunction and Exclusion (TIDE) algorithm [21], based on lung adenocarcinoma RNAseq data from TCGA database. TISIDB (http://cis.hku.hk/TISIDB/, accessed on 1 June 2022) [22] is a comprehensive repository portal for tumor immune system interactions, which is based on the publicly available PubMed literature data for deep analysis. We explored the relationship between CDK1 and immune regulators using TISIDB and found that CDK1 was closely related to the IL family. Further, we collected a total of 17 patients who were hospitalized and diagnosed with lung adenocarcinoma by pathological biopsy in the First Affiliated Hospital of Chongqing Medical University. IL levels were measured before and after immunotherapy. In addition, we judged the patients’ positive response and negative response to immunotherapy by iRECIST criteria.

#### 2.3.2. Immune Infiltration Analysis

RNAseq data and clinical information of lung adenocarcinoma were obtained from TCGA database. The ssGSEA algorithm was used to calculate the immune cell infiltration scores of the samples. When intergroup comparisons were performed, Student’s t-test was used if the data met the requirements of normality and homogeneity of variance. In addition, Welch's t-test was used if two groups of data only met the requirements of normality but not homogeneity of variance. Moreover, when the two groups of data do not conform to normality, the Wilcoxon rank sum test was used for testing. The above results were visualized with the ggplot2 package of R software.

#### 2.3.3. Clinical Relevance Analysis

Patients with lung adenocarcinoma in TCGA database were grouped according to the expression of target protein, and the correlation with clinical stage, gender, age, race, smoking, and other conditions was analyzed by chi-squared test and *t*-test.

### 2.4. Pathway Correlation Analysis of Molecular

We collected the set of genes contained in relevant pathways [23] and obtained RNAseq data and corresponding clinical information of lung adenocarcinoma from the TCGA database. Next, analysis was performed by the R software GSVA package, according to the ssGSEA algorithm [24]. An enrichment score was calculated for each sample on each pathway in turn. Later, we analyzed the correlation of genes with pathway scores by spearman correlation. On the other hand, Metascape is a gene-annotation and gene-list-enrichment analysis database and tool (https://metascape.org/, accessed on 5 June 2022) [25]. To further explore the differential gene roles, we obtained co-expressed genes simultaneously associated with CDK1 and CXCL8 by single-gene differential expression analysis. In addition, we imported the co-expressed genes to Metascape tool, built protein–protein interaction network (PPI), and used Metascape's online analysis function to perform gene ontology (GO) analysis and visualize the DEGs from biological process, molecular function, and KEGG pathway, respectively.

### 2.5. Single-Cell Analysis

Human Protein Atlas database (https://www.proteinatlas.org/, accessed on 6 June 2022) is dedicated to providing tissue and cellular distribution information of various classes of human proteins, and we derived RNA expression of single-cell-type clusters in lung tissue with its help. Cancer Single-cell Expression Map (https://ngdc.cncb.ac.cn/cancerscem/, accessed on 6 June 2022) [26] is a public database dedicated to collecting, analyzing, and visualizing single-cell RNA-Seq data from human cancers. We explored the single-cell expression landscape of target molecules using its online analysis part. CANCERSEA is an additional single-cell database (http://biocc.hrbmu.edu.cn/CancerSEA/home.jsp, accessed on 10 June 2022) [27], which aims to comprehensively decode distinct functional states of cancer cells at single-cell resolution. It can provide PCG/lncRNA repertoires that are highly related to functional states at single-cell resolution. We searched the correlation between RNAs and functional states through this database.

## 3. Results

### 3.1. Clinically Relevant Information for CDK1

**Figure 1:**
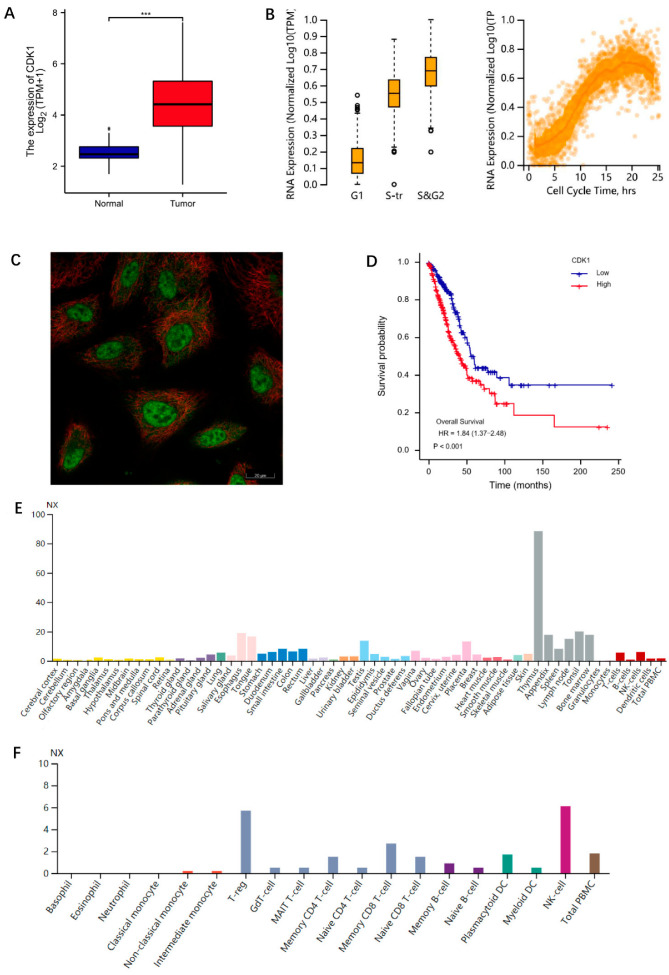
Information for CDK1. (**A**) CDK1 expression in normal tissues and LUAD. Significance markers: *** *p* < 0.001. (**B**) The expression of CDK1 changed during the cell cycle. (**C**) The localization of CDK1 in A549 cell. Green for CDK1 antibody; red for tubulin. (**D**) Correlation between CDK1 expression and OS of LUAD patients. (**E**) Tissue-specificity analysis. (**F**) The results of CDK11 expression levels in blood cell samples. The analysis data of (**A**,**D**) were from TCGA database. The analysis data of (**B**,**C**,**E**,**F**) were from HPA database.

Gene expression data of lung adenocarcinoma patients in the TCGA database were downloaded (https://portal.gdc.cancer.gov/, accessed on 17 March 2022), a total of 59 normal tissues and 539 lung adenocarcinoma tissues were included (the sample ID associated with the study in the TCGA database is provided in Supplementary Table S1), and the expression of CDK1 in normal tissues was statistically compared with that in lung adenocarcinoma tissues. The results showed that CDK1 expression was significantly higher in tumor tissues compared with normal tissues (Figure 1A). We further explored some information about CDK1 with the Human Protein Atlas database. The results showed that the expression of CDK1 changed according to the cell cycle (Figure 1B) and was at a low value in G1 phase, followed by a gradual rise and a peak in the S and G2 phases. Following the G2 phase, CDK1 expression gradually declined again. It is acknowledged that cell growth divisions must sequentially go through the pre-phase interphase and mitotic phase. The activity of interphase (including the G1, S, and G2 phases) provides each intracellular component required for division in the M phase, and the end of each mitosis to the end of the next one constitutes a complete cell cycle. This result further verified that CDK1 played an important role in cell cycle regulation. Tissue specificity analysis found (Figure 1E) that CDK1 was enriched in lymphoid tissue, especially in the thymus, suggesting that CDK1 may be involved in some immune regulation and mechanisms. The results of mRNA expression levels in blood cell samples suggested that CDK1 was related to the enrichment of immune cells (Figure 1F), especially NK cells and T-regs. We further explored the localization of CDK1 in cells, and the results suggested that CDK1 is mainly locked in nucleoplasm and cytosol (Figure 1C). The prognostic value of CDK1 was further explored by clinically relevant information in lung adenocarcinoma from TCGA database, the survival package was used for statistical analysis of survival data, and the survminer package was used for visualization. The results indicated that the median survival time of the CDK1 low expression group was 59.3 months, whereas that of the CDK1 high expression group was only 41 months (Figure 1D). The general information of patients with different CDK1 expression levels was shown in Table 1. This phenomenon suggested that high CDK1 expression was a relevant factor for poor prognosis in lung adenocarcinoma. Therefore, screening the related pathway molecules that target CDK1 might be a potential effective way of research in lung adenocarcinoma prevention and treatment.

### 3.2. Exploration of CDK1-Associated lncRNAs

#### 3.2.1. Exploration of CDK1-Associated DEGs

**Figure 2:**
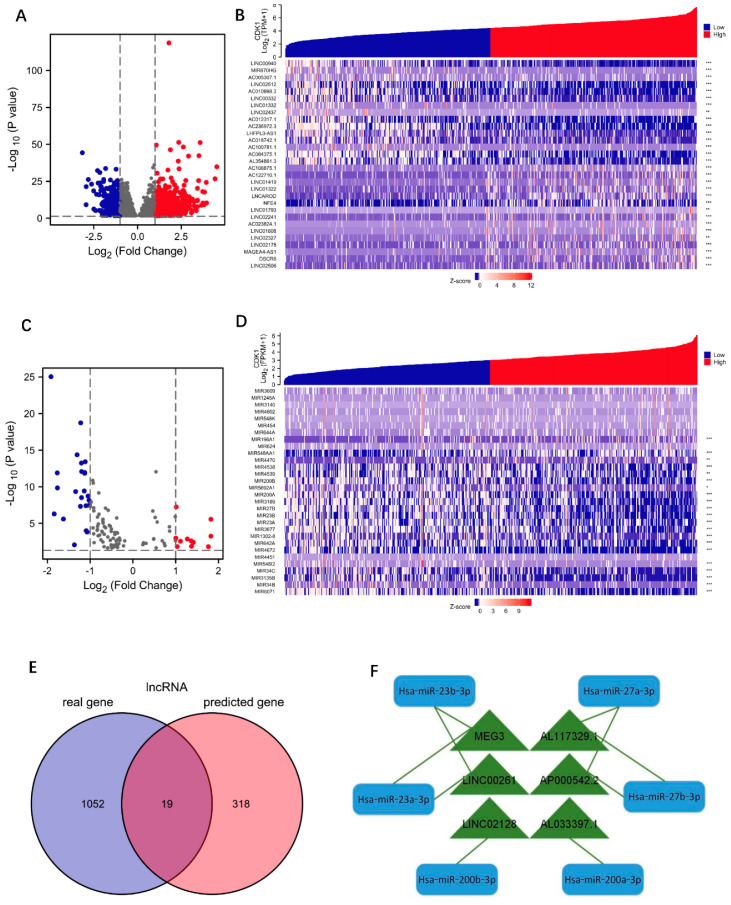
Exploration of CDK1-associated DEGs. (**A**) The volcano plots describe DElncRNAs. log_2_ foldchange > 1 or log_2_ foldchange < −1, * *p* < 0.05. (**B**) CDK1-associated co-expression heatmap with the top 30 DElncRNAs. Significance markers: ** *p* < 0.01; *** *p* < 0.001. (**C**) The volcano plots describe DEmiRNAs. log_2_ foldchange > 1 or log_2_ foldchange < −1, * *p* < 0.05. (**D**) CDK1-associated co-expression heatmap with the top 30 DEmiRNAs. Significance markers: ** *p* < 0.01; *** *p* < 0.001. (**E**) The lncRNAs related to CDK1 are represented by Venn diagram. (**F**) CDK1-associated lncRNA-miRNA regulatory network.

LUAD RNAseq data were extracted from the TCGA database. A total of 56,493 DEGs associated with CDK1 was found by single-gene differential-expression analysis, which included 1448 differentially expressed miRNAs as well as 14,077 differentially expressed lncRNAs. Statistically significant DEGs were further selected, and 1071 lncRNAs and 34 miRNAs met the screening requirements (Figure 2A,C). Next, the top 30 DEmiRNAs and DElncRNAs with the largest expression difference were selected to draw the co-expression heat maps related to CDK1, respectively. The results were presented in Figure 2B,D.

#### 3.2.2. Identification of Hub-lncRNA

**Figure 3:**
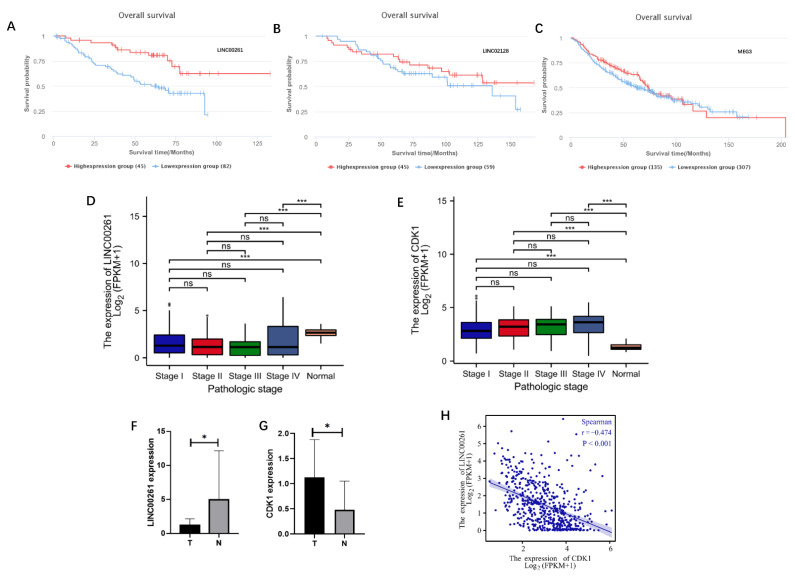
Identification of Hub-lncRNA. (**A**–**C**) Survival analysis of lncRNAs in LUAD. The data are from the LUAD project in TCGA database. (**A**) LINC00261, (**B**) LINC02128, and (**C**) MEG3. (**D**,**E**) LINC00261 (**D**) and CDK1 (**E**) expression in normal tissues and LUAD with different stages. The data for analysis were from the LUAD project in TCGA database. Significance markers: ns, *p* ≥ 0.05; * *p* < 0.05; *** *p* < 0.001. (**F**,**G**) The expression differences of LINC00261 (**F**) and CDK1 (**G**) in LUAD and normal tissues were verified by RT-qPCR. Significance markers: ns, *p* ≥ 0.05; * *p* < 0.05; *** *p* < 0.001. (**H**) Correlation analysis of CDK1 and LINC00261.

ENCORI website is an open platform for investigating the interaction between molecules (http://starbase.sysu.edu.cn/index.php, accessed on 17 March 2022) [18], which identifies more than 1.1 million miRNA–lncRNA interactions from multi-dimensional sequencing data. Excluding the currently unreported miRNA prediction results, a total of 12 miRNAs were taken to correspond to a total of 337 predicted lncRNAs. The predicted RNA molecules and the actual differentially expressed RNA molecules obtained from data analysis were then intersected in the Venn diagram. In addition, a total of 19 lncRNAs were subsequently found (Figure 2E). These 19 lncRNAs and DEmiRNAs were integrated into lncRNA–miRNA pairs, using the principle of base complementary pairing, to construct an lncRNA-miRNA regulatory network (Figure 2F), which included six lncRNAs (MEG3, AP00542.2, LINC00261, AL117329.1, LINC02128, and AL033379.1) and five miRNAs (Hsa-miR-23b-3p, Hsa-miR-23a-3p, Hsa-miR-27b-3p, Hsa-miR-27b-3p, Hsa-miR-200b-3p, and Hsa-miR-200a-3p). Survival analysis found that lung cancer patients with low LINC00261 expression had a significantly better prognosis than those with high LINC00261 expression (Figure 3A), while the other two lncRNAs (LINC02128 and MEG3) did not show significant correlation with survival (Figure 3B,C). The remaining three lncRNAs (AP00542.2, AL117329.1, and AL033379.1) currently lack clinical survival conclusions.

#### 3.2.3. Validation of RNA Expression Levels and Clinical Relevance

TCGA data validated that LINC00261 expression in normal tissues was significantly higher than that in tumor tissues (Figure 3D). Meanwhile, CDK1 expression in normal tissues was significantly lower than that in tumor tissues (Figure 3E). Further, lung adenocarcinoma patients from TCGA database were grouped according to LINC00261 expression, and the correlations between LINC00261 and clinical stage, gender, age, race, and smoking were analyzed. The results are shown in Table 2. There was no significant difference in LINC00261 expression with different tumor stages, gender, age, and race (*p* > 0.05). However, non-smokers expressed higher LINC00261 than smokers, which was statistically significant (*p* = 0.019). At the same time, we collected six post-operative samples of lung adenocarcinoma from the First Affiliated Hospital of Chongqing Medical University, measured the expression of LINC00261 and CDK1 in tumor tissues and adjacent noncancerous tissues by RT-qPCR, respectively (the data of one patient was discarded due to internal reference error), and performed comparative analysis; the results are shown in Figure 3F,G. The basic information of the patients is shown in Supplementary Table S2.

#### 3.2.4. Molecular Correlation Analysis

The results of molecular correlation analysis suggested that there was a significant negative correlation between LINC00261 and CDK1 in lung adenocarcinoma samples (Figure 3H). Combined with the results of survival analysis and other comprehensive analysis, it suggested that there might be a regulatory relationship between LINC00261 and CDK1, so loss of LINC00261 in lung adenocarcinoma patients might lead to CDK1 overexpression.

### 3.3. Immunotherapy Response Modulation Explorations

#### 3.3.1. Prediction of Responsiveness to ICIs

**Figure 4:**
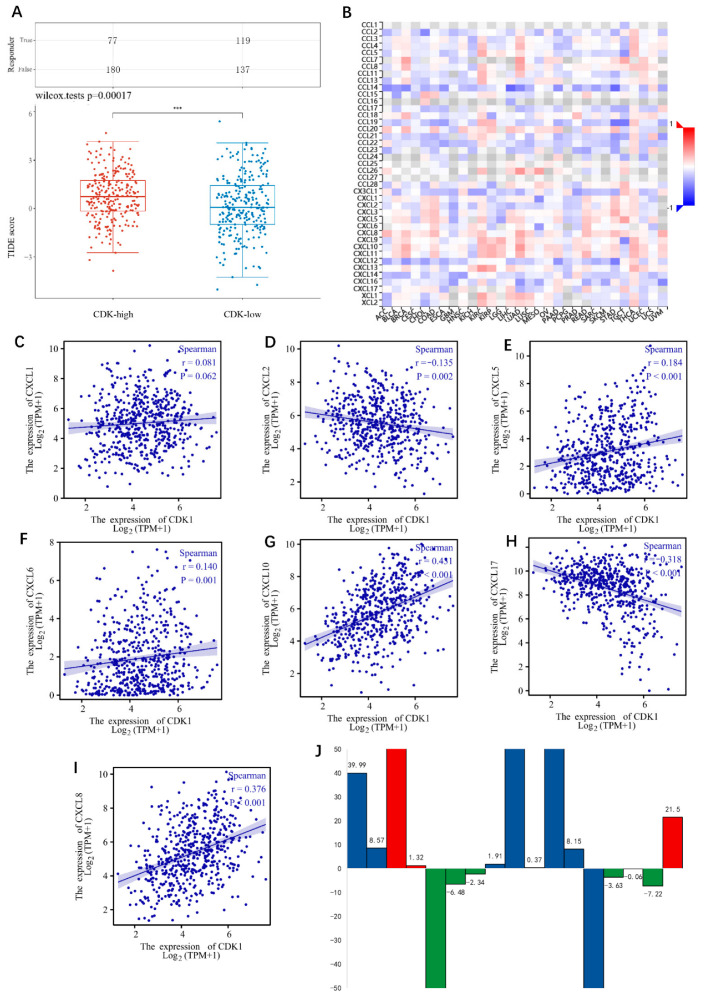
(**A**) The correlation between the expression of CDK1 and the response of immune checkpoint inhibitors. *** *p* < 0.001. (**B**) The correlation heatmap between CDK1 and immune regulatory factors drew by TISIDB. (**C**–**I**) Correlation analysis of CDKs and CXCLs; (**C**) CDK1 and CXCL1; (**D**) CDK1 and CXCL2; (**E**) CDK1 and CXCL5; (**F**) CDK1 and CXCL6; (**G**) CDK1 and CXCL10; (**H**) CDK1 and CXCL17; (**I**) CDK1 and CXCL8. The data for analysis were from the LUAD project in TCGA database. (**J**) Fluctuation of IL8 in peripheral blood during immunotherapy in 17 lung adenocarcinoma patients. Red represents immunotherapy resistant population; green represents the immunotherapy effective population; blue represents the population in which immunotherapy efficacy could not be judged.

The Tumor Immune Dysfunction and Exclusion (TIDE) algorithm [21] was used to predict the correlation between the expression of CDK1 and the response of immune checkpoint inhibitors. TIDE uses a set of gene expression markers to evaluate two different mechanisms of tumor immune escape, including the dysfunction of tumor infiltrating cytotoxic T lymphocytes (CTLs) and the rejection of CTLs by immunosuppressive factors. The higher the TIDE score is means the worse the effect of ICIs and the shorter the survival time. The analysis results showed that the TIDE score in the CDK1 high expression group was higher than that in the low expression group (Figure 4A), suggesting that the high expression of CDK1 might mean the poor effect of immunotherapy.

#### 3.3.2. CDK1 Was Closely Related to CXCL Family

In order to understand the mechanism of immune escape, we used TISIDB database to explore the relationship between CDK1 and immune regulatory factors and found that CDK1 was closely related to the CXCL family (Figure 4B). Further analysis of the correlation between CDK1 and CXCL molecules using the TCGA database is shown in Figure 4C–I. CDK1 showed no obvious correlation with CXCL1, while CDK1 was positively correlated with CXCL5, CXCL6, CXCL8, and CXCL10 and negatively correlated with CXCL1 and CXCL17. Among those, CXCL8 and CXCL10 were more frequently associated with CDK1.

#### 3.3.3. Relevance of the IL Family to Immunotherapy

Current studies have found that the IL family is associated with the efficacy of immunotherapy [28,29,30], and some molecules of the IL family have been studied and found to serve as predictors of the efficacy of immunotherapy [31]. In addition, our study also found that CDK1 was closely related to CXCLs. To explore the correlation between the IL family and immunotherapy in lung adenocarcinoma patients, we collected peripheral blood samples from 17 lung adenocarcinoma patients who had received immunotherapy and measured the ILs levels before and after immunotherapy. The basic information of patients is shown in Supplementary Table S3. The positive response and negative response of patients to immunotherapy were judged by iRECIST criteria. The ILs monitoring level result is shown in Table 3. The results suggested that in patients receiving immunotherapy, IL8 values fluctuated more significantly during immunotherapy than those of other molecules of the IL family. The six patients who exhibited a response to immunotherapy had a decline in peripheral blood IL8. The three patients exhibiting no response to immunotherapy had a rise in peripheral blood IL8. The other eight patients could not be judged on their immunotherapy response, because they were in a tumor-free state after surgery or had co-infectious factors. In general, there were seven patients who experienced a decline in IL8 during immunotherapy, six of whom had an immunotherapy response. Meanwhile, there were 10 patients who presented with a rise in IL8, 3 of whom developed immune resistance. The result is shown in Figure 4J.

#### 3.3.4. Clinical Relevance of CXCL8

Lung adenocarcinoma patients from TCGA database were grouped according to CXCL8 expression, and the correlations between LINC00261 and clinical stage, gender, age, race, and smoking were analyzed; the result is shown in Table 4. There was no significant difference in CXCL8 expression among patients with different T stages and M stages. However, CXCL8 expression was higher in patients with lymph node metastasis than in the patients without lymph node metastasis. It suggested that the increase in CXCL8 might increase the frequency of lymph node metastasis. In addition, gender, age, race, and smoking did not show a significant correlation with CXCL8.

#### 3.3.5. Immune Infiltration Analysis

**Figure 5:**
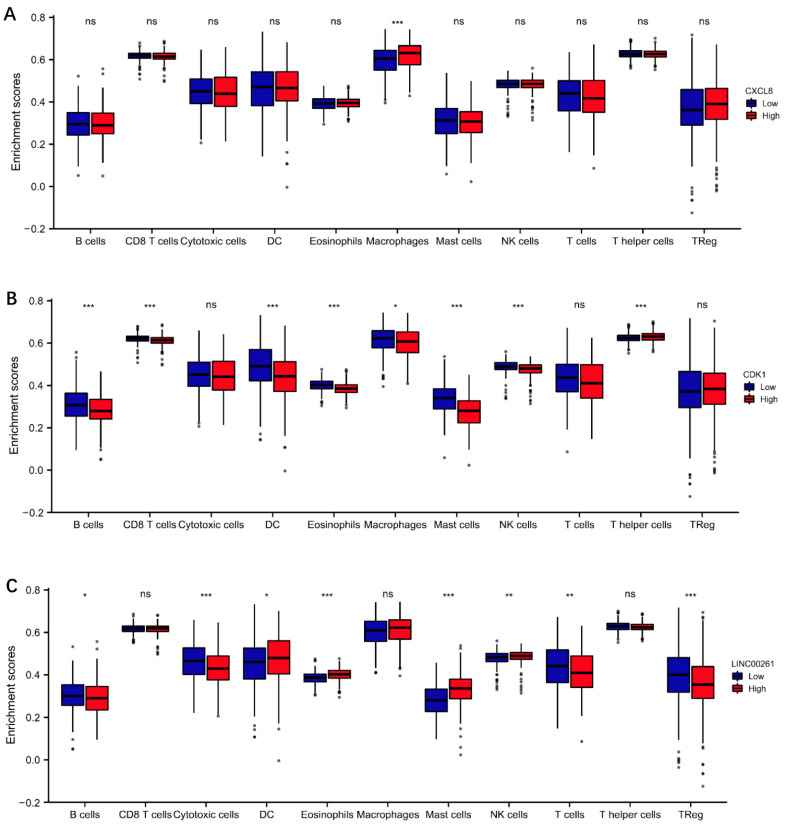
Immune infiltration analysis. (**A**) CXCL8. (**B**) CDK1. (**C**) LINC00261. Significance markers: ns, *p* ≥ 0.05; * *p* < 0.05; ** *p* <0.01; *** *p* < 0.001. The data for analysis are from the LUAD project in TCGA database.

The immune infiltration analysis result showed that CXCL8 was mainly associated with macrophages. CXCL8 high expression suggested more macrophage infiltration, as shown in Figure 5A. In addition, high expression of CXCL8 was also associated with increased Treg cell infiltration, but the results were not statistically significant. However, CXCL8 expression did not seem to be significantly associated with B cells, T cells, mast cells, Th cells, or NK cells. CDK1 high expression suggested lower DC cell infiltration, mast cell infiltration, CD8+ T cell infiltration, macrophage infiltration, and NK cell infiltration. However, cytotoxic T cell infiltration was not significantly correlated with the CDK1 expression situation (Figure 5B). LINC00261 high expression suggested lower Treg infiltration and more cytotoxic T cell infiltration (Figure 5C).

### 3.4. Pathway Correlation Analysis of Molecular

**Figure 6:**
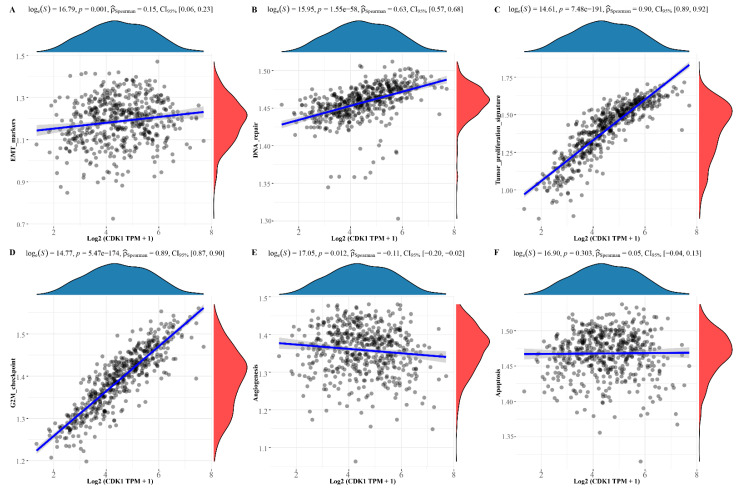
Pathway correlation analysis of CDK1. (**A**) EMT makers. (**B**) DNA repair. (**C**) Tumor proliferation. (**D**) G2-M checkpoint. (**E**) Angiogenesis. (**F**) Apoptosis.

**Figure 7:**
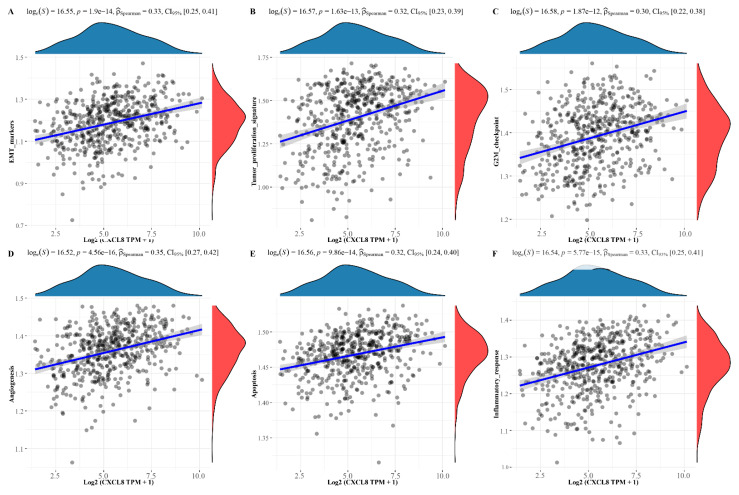
Pathway correlation analysis of CXCL8. (**A**) EMT makers. (**B**) Tumor proliferation. (**C**) G2-M checkpoint. (**D**) Angiogenesis. (**E**) Apoptosis. (**F**) Inflammatory response.

**Figure 8:**
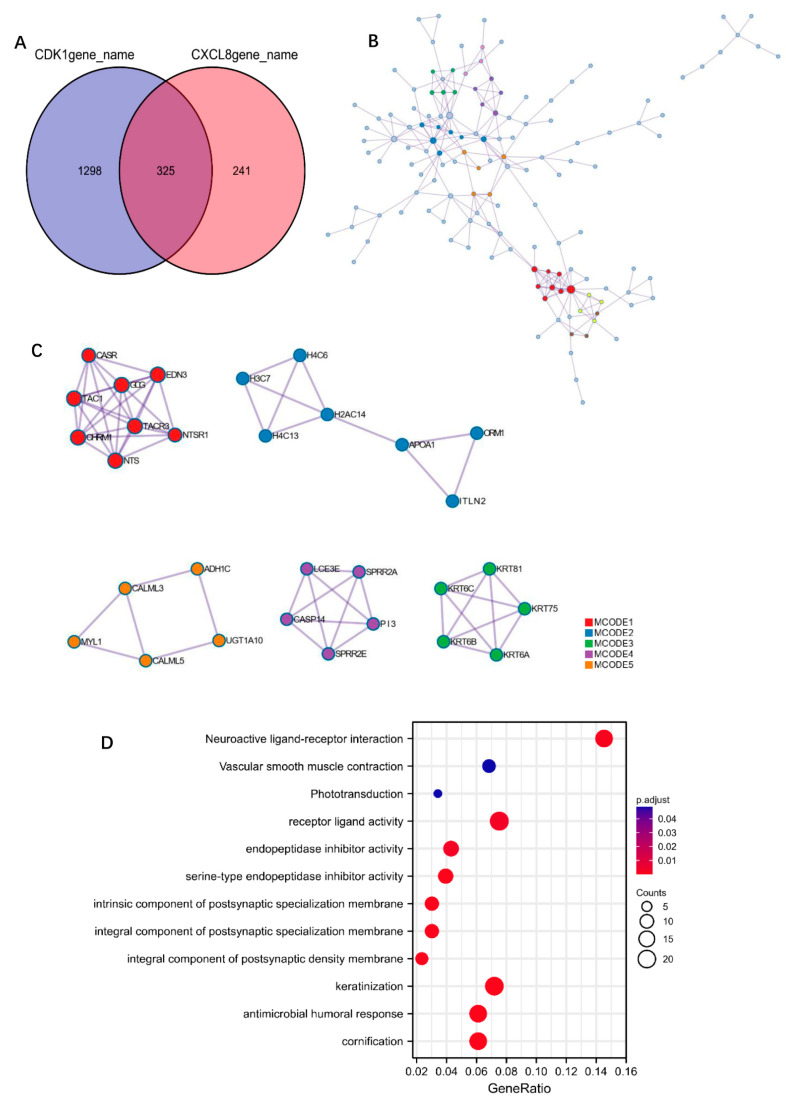
Enrichment analysis of CDK1 and CXCL8. (**A**) Venn diagram of the co-expressed genes related to CDK1 and CXCL8. (**B**) The PPIs of the co-expressed genes. (**C**) MCODE part of the PPIs. (**D**) Enrichment analysis results of co-expressed genes.

We separately assessed some common functional pathways by ssGSEA algorithm, including: PI3K/Akt/mTOR pathway, cellular hypoxia response, EMT-related genes, G2M checkpoint, angiogenesis, tumor proliferation, and so on. The results suggest that CDK1 was positively correlated with PI3K/Akt/mTOR pathway, cellular hypoxia response, EMT related genes, G2M checkpoint, and tumor proliferation, while negatively correlated with angiogenesis. Among them, CDK1 showed a higher correlation index with the G2M checkpoint, tumor proliferation, which is shown in Figure 6A–F. Meanwhile, CXCL8 was positively correlated with tumor proliferation, EMT, angiogenesis, and the IL10 inflammasome pathway; the results are shown in Figure 7A–F. To understand the potential common pathway between CDK1 and CXCL8, 325 co-expressed genes of CDK1 and CXCL8 were obtained by differential expression analysis (Figure 8A), and, next, the protein-interaction-network diagram was obtained by using Metascape’s online analysis function (Figure 8B). The MCODE algorithm [32] was applied to identify the densely connected networks, and the results are shown in Figure 8C. Go enrichment analysis was performed for each MCODE network, which is described in Table 5. In addition, enrichment analysis of all the co-expressed genes is shown in Figure 8D.

### 3.5. Single-Cell Analysis

**Figure 9:**
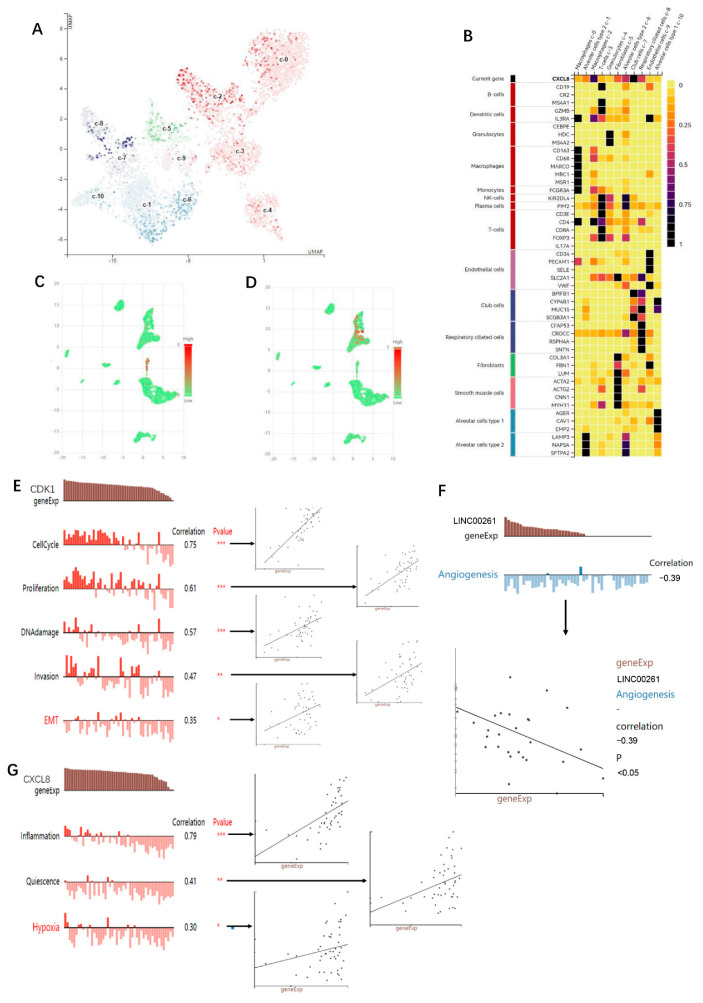
Results of single cell analysis. (**A**) CXCL8 expression in normal lung tissues. (**B**) Cell maker of the cells in (**A**). The analysis of (**A**,**B**) are from HPA database. (**C**) Single cell analysis of CDK1 expression in lung cancer tumor tissues. (**D**) Single cell analysis of CXCL8 expression in lung cancer tumor tissues. The analysis of (**C**,**D**) are from Cancer Single-cell Expression Map database. (**E**) Functional states analysis of CDK. (**F**) Functional states analysis of LINC00261. (**G**) Functional states analysis of CXCL8. The analysis of (**E**–**G**) are from CANCERSEA database. Significance markers: * *p* < 0.05; ** *p* <0.01; *** *p* < 0.001.

The results of single cell analysis suggested that in normal lung tissue, CXCL8 was more abundantly expressed in macrophages (Figure 9A). The respective cellular makers are expressed in Figure 9B. Cancer single cell expression map database analysis indicated that in tumor tissues, CDK1 was mainly expressed in macrophages, followed by DC cells. In addition, a little CDK1 expression was observed on CD8+ T cells and fibroblasts (Figure 9C). CXCL8 was mainly expressed in macrophages; in addition, there was a little expression on CD8+ naïve T cells, fibrocytes, NK cells, and mast cells (Figure 9D). The results of functional states analysis suggested that LINC00261 was mainly negatively correlated with angiogenesis (Figure 9F). CDK1 was significantly correlated with cell cycle, proliferation, DNA damage, invasion, and EMT (Figure 9E). Meanwhile, CXCL8 showed a significant positive correlation with inflammation, quiescence, and hypoxia (Figure 9G).

**Figure 10:**
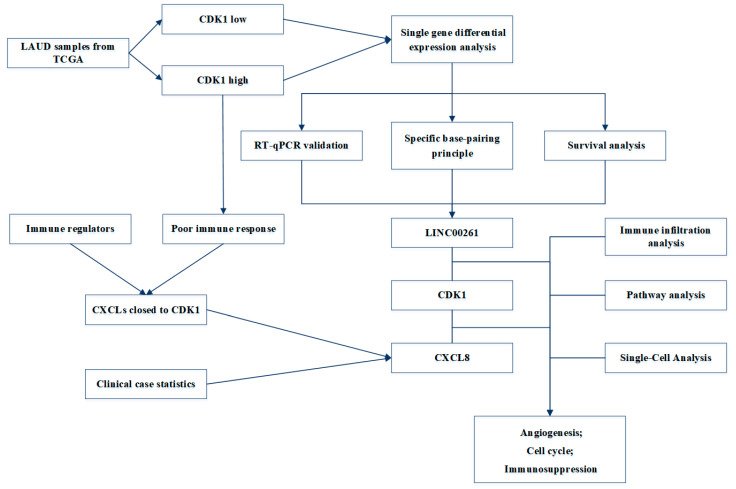
Flowchart of the study.

In order to visually show the research process and main results of this study, we have drawn a flowchart, as shown in Figure 10.

## 4. Discussion

CDK inhibitors have always been the focus of clinical drug research and development. However, only a CDK4/6 inhibitor is used in the clinical treatment of malignant tumors [9], and the exact efficacy and safety of other CDK inhibitors are still uncertain. During the cell cycle, there are two checkpoints that prevent cells from repairing damaged DNA to maintain genomic integrity. The ATM-CHK2-p53 pathway controls the G1 checkpoint, while the ATR-CHK1-Wee1 pathway controls the S and G2/M checkpoints [10]. Cyclin A and CDK1 complex participate in the S–G2 phase transition, while cell cycle B participates in the G2–M phase transition through the CDK1 complex [11]. Preclinical studies have found that anti-CDK1 treatment can promote the antitumor response of sorafenib in hepatocellular carcinoma [12]. However, in recent years, drug trials that directly target CDK1 have been in the early stage of clinical trials without any breakthrough progress, suggesting that there may be some difficulties in directly targeting CDK1.

Our study found that LINC00261 is an important lncRNA closely related to CDK1. The transcription site of LINC00261 is located at 22,560,552–22,578,642 on chromosome 20 [33]. Overexpression of LINC00261 inhibits cell proliferation and invasion and promotes apoptosis in non-small cell lung cancer cell lines [34]. Our study also confirmed that the expression of LINC00261 decreased significantly in LUAD, and lower expression predicted a worse prognosis. It is suggested that LINC00261, as a tumor suppressor, may be a potential biomarker or therapeutic target. Based on the correlation analysis of clinical data, we found that LINC00261 expression was higher in non-smokers, suggesting that smoking may be an important factor regulating the expression of LINC00261. Reviewing the current research on lung cancer, we found that LINC00261 may participate in tumor progression through the following mechanisms: (1) miR-522-3p, Wnt pathway [34]; (2) miR-105/FHL1 axis [35]; (3) FOXA2, ERK pathway [36]; and (4) miR-1269a/FOXO1 axis [37]. It has also been found that targeted demethylation of the LINC00261 promoter inhibited pancreatic cancer progression [38]. Our results suggest that the decreased expression of LINC00261 promotes the overexpression of CDK1, further promotes the occurrence of lung adenocarcinoma, and affects the prognosis of patients. LINC00261 may serve as a key target in the research progress of anti-CDK1 pathway.

The IL family is currently considered to affect the development of tumor in tumor microenvironment through at least two pathways [39]: (1) cytokines affect the proliferation/apoptosis of tumor cells and; (2) drive antitumor immune activity. The study found that during the immunotherapy of lung cancer patients, the serum IL-8 level decreased significantly in the responding patients. Meanwhile, the level of IL-8 increased significantly among non-responders. It shows that the changes of serum IL-8 level are closely related to the efficacy of immunotherapy. IL-8 is mainly encoded by CXCL8 and has been confirmed to be mainly secreted by macrophages. Activation of M1 macrophages increases their ability to present antigens and kill pathogens through major histocompatibility complex (MHC) class II molecules. On the other hand, the increase in M2 macrophage infiltration is currently considered to be associated with adverse clinical outcomes of cancer [30]. Through single cell analysis, we found that CXCL8 was mainly expressed in macrophages in lung adenocarcinoma samples, suggesting that CXCL8 may induce an immunosuppressive microenvironment through macrophages, thereby promoting tumor progression.

In addition, this study found that high expression of CDK1 was often associated with poor response to immunotherapy. On the one hand, the expression of LINC00261 in lung cancer tissues was significantly lower than that in normal tissues. Low expression of LINC00261 suggests higher Treg infiltration and less cytotoxic T cell infiltration. The immune microenvironment under immunosuppression may explain the high TIDE score. We suspect that increasing the expression of LINC00261 may contribute to the release of Treg immunosuppression and improve the response rate of immunotherapy. On the other hand, there is a significant positive correlation between the expression of CDK1 and CXCL8 in patients with lung adenocarcinoma, suggesting that CDK1 may have a regulatory relationship with CXCL8. In addition, this study found, through the analysis of clinical samples, that IL-8 tended to be elevated in the peripheral blood of immunotherapy-resistant patients . While in patients who responded to immunotherapy, the IL-8 levels were declining. It is suggested that increased IL-8 may indicate the occurrence of immunotherapy resistance. We hypothesized that if regulated at the level of LINC00261 and CDK1, inhibiting IL-8 secretion and remodeling the tumor immune microenvironment might reverse immune resistance and improve the effectiveness of lung adenocarcinoma patients receiving immunotherapy.

To explore the potential relationship between CDK1 and CXCL8, we separately assessed the links between CDK1/CXCL8 and some common functional pathways. It was found that CDK1 together with CXCL8 participate in G2M checkpoint, tumor proliferation, EMT, and other pathways. In addition, we obtained co-expressed mRNAs with CDK1 and CXCL8, which participated in biological processes and molecular functions including antimicrobial humoral response, receptor-like activity, and so on. In addition, hub gene functions mainly focus on G protein signaling pathway, RNA polymerase I promoter, and DNA methylation. A review of the literature indicates that CXCL8 may be a mitotic target of the CDK1/CDC14B-USP9X-WT1 signaling axis, which conveys mitotic survival [40]. Inhibition of CDK1 may inhibit transcription factor WT1 deubiquitination and stabilization, which, in turn, inhibits mitosis-specific transcriptional activation of CXCL8, with a consequent reduction in mitotic survival through reduced IL-8 expression and secretion. The function of CXCL8 was confirmed to be mainly dependent on its interaction with specific cell-surface G protein-coupled receptors (GPCRs), CXCR1, and CXCR2 [41]. The mechanisms of CXCL8-CXCR1/2 signaling in tumorigenesis and tumor progression have been extensively explored. Additionally, autocrine CXCL8 and VEGF synergistically mediated neovascularization and EMT, thereby promoting the invasion of A549 cells [42]. This conclusion was also validated in our analysis. Moreover, our results found that the pathway exploration of LINC00261 suggested a significant negative correlation with angiogenesis. This result suggested that elevating LINC00261 expression in tumors might also inhibit CXCL8-mediated tumor angiogenesis.

## 5. Conclusions

In this study, we validated, by bioinformatics analysis, a basic experiment, and clinical case analysis, that all LINC00261, CDK1, and CXCL8 may play important roles in the development and progression of lung adenocarcinoma, response to treatment effects that involved angiogenesis, cell cycle phase transition, and immune-related pathways. This study provides new ideas for translational research on CDK1. Further investigation of these interactions, to optimize therapeutic targets, may improve the therapeutic limitations for NSCLC with a negative driver gene.

## Figures and Tables

**Table 1:** The general information of patients with different CDK1 expression levels. The data for analysis were from the LUAD project in TCGA database.

Characteristic	Low Expression of CDK1	High Expression of CDK1	*p*
n	256	257	
T stage, n (%)			<0.001
T1	108 (21.2%)	60 (11.8%)	
T2	115 (22.5%)	161 (31.6%)	
T3	23 (4.5%)	24 (4.7%)	
T4	8 (1.6%)	11 (2.2%)	
N stage, n (%)			<0.001
N0	184 (36.7%)	146 (29.1%)	
N1	34 (6.8%)	61 (12.2%)	
N2	29 (5.8%)	45 (9%)	
N3	0 (0%)	2 (0.4%)	
M stage, n (%)			0.023
M0	171 (46.3%)	173 (46.9%)	
M1	6 (1.6%)	19 (5.1%)	
Gender, n (%)			0.009
Female	153 (29.8%)	123 (24%)	
Male	103 (20.1%)	134 (26.1%)	
Race, n (%)			0.967
Asian	4 (0.9%)	3 (0.7%)	
Black or African American	28 (6.3%)	24 (5.4%)	
White	200 (44.8%)	187 (41.9%)	
Age, n (%)			0.151
≤65	111 (22.5%)	127 (25.7%)	
>65	137 (27.7%)	119 (24.1%)	
Smoker, n (%)			0.105
No	44 (8.8%)	30 (6%)	
Yes	206 (41.3%)	219 (43.9%)	

**Table 2:** Clinical information on LINC00261. The data for analysis were from the LUAD project in TCGA database.

Characteristic	Low Expression of LINC00261	High Expression of LINC00261	*p*
n	256	257	
T stage, n (%)			0.223
T1	73 (14.3%)	95 (18.6%)	
T2	146 (28.6%)	130 (25.5%)	
T3	26 (5.1%)	21 (4.1%)	
T4	9 (1.8%)	10 (2%)	
N stage, n (%)			0.324
N0	162 (32.3%)	168 (33.5%)	
N1	45 (9%)	50 (10%)	
N2	42 (8.4%)	32 (6.4%)	
N3	2 (0.4%)	0 (0%)	
M stage, n (%)			0.899
M0	177 (48%)	167 (45.3%)	
M1	12 (3.3%)	13 (3.5%)	
Gender, n (%)			0.454
Female	133 (25.9%)	143 (27.9%)	
Male	123 (24%)	114 (22.2%)	
Race, n (%)			0.435
Asian	2 (0.4%)	5 (1.1%)	
Black or African American	28 (6.3%)	24 (5.4%)	
White	187 (41.9%)	200 (44.8%)	
Age, n (%)			0.855
≤65	117 (23.7%)	121 (24.5%)	
>65	129 (26.1%)	127 (25.7%)	
Smoker, n (%)			0.019
No	27 (5.4%)	47 (9.4%)	
Yes	221 (44.3%)	204 (40.9%)	

**Table 3:** Changes in ILs before and after immunotherapy.

	ILs	IL-2		IL-4		IL-5		IL-6		IL-8		IL-10		IL-17	
Patients		Before	After	Before	After	Before	After	Before	After	Before	After	Before	After	Before	After
1		0.4	0.22	0.38	0.92	8.37	0.82	0.54	3.43	0.38	40.37	0.72	0.24	0.1	0.59
2		0.23	0.4	0.46	0.13	0.11	1.08	0.44	0.57	0.61	9.18	0.19	0.47	0.97	1.52
3		0.35	0.11	0.82	0.39	0.14	5.12	0.65	0.43	33.09	91.08	0.19	2.18	0.85	1.83
4		1.71	0.31	0.6	0.13	0.32	2.53	5.19	0.17	4.09	5.41	0.68	0.58	3.11	0.55
5		0.18	0.3	0.66	0.24	0.51	0.29	1.51	0.11	245.86	15.39	0.79	0.7	3.93	0.2
6		0.11	0.3	0.41	0.18	3.44	0.45	9.22	0.36	7.18	0.7	1.06	0.7	3.32	0.2
7		0.4	0.3	0.22	0.21	0.5	1.13	8.72	0.15	6.63	4.29	0.19	0.58	0.16	0.2
8		1.72	0.7	0.44	0.45	9.25	1.59	3.85	0.53	0.9	2.81	0.28	0.96	0.17	2.23
9		0.62	9.33	2.11	1.97	0.21	18.99	18.18	31.8	38.64	136.39	2.01	14.52	0.17	13.1
10		0.35	0.7	0.22	0.13	0.55	1.48	0.61	1.88	0.25	0.62	0.12	0.34	0.17	1.75
11		0.15	0.37	0.29	0.27	0.52	0.85	0.29	0.22	30.73	143.97	0.53	0.76	0.55	0.4
12		0.74	0.15	1.07	0.32	0.49	0.38	2.28	0.13	0.27	8.42	0.89	1.15	1.04	0.55
13		0.15	0.37	0.46	0.27	3.89	0.7	1.03	0.22	157.41	12.37	7.6	0.2	0.72	0.4
14		0.97	0.27	0.53	0.1	4.07	4.66	2.88	0.35	5.61	1.98	0.74	0.89	1.78	0.13
15		1.26	0.85	0.7	0.11	4.44	0.56	11.5	0.19	0.25	0.19	0.61	0.46	1.45	0.22
16		0.45	0.28	0.44	1.21	3.12	0.07	2.42	1	7.65	0.43	0.85	0.69	0.84	0.83
17		0.33	0.13	0.94	0.43	0.32	1.81	1.93	3.82	1.47	22.97	0.56	0.33	0.04	0.45
Reference (pg/mL)		<7.5		<8.56		<3.1		<5.4		<20.6		<12.9		<21.4	

**Table 4:** Clinical information on CXCL8. The data for analysis were from the LUAD project in TCGA database.

Characteristic	Low Expression of CXCL8	High Expression of CXCL8	*p*
n	256	257	
T stage, n (%)			0.081
T1	93 (18.2%)	75 (14.7%)	
T2	129 (25.3%)	147 (28.8%)	
T3	20 (3.9%)	27 (5.3%)	
T4	13 (2.5%)	6 (1.2%)	
N stage, n (%)			0.002
N0	183 (36.5%)	147 (29.3%)	
N1	39 (7.8%)	56 (11.2%)	
N2	26 (5.2%)	48 (9.6%)	
N3	1 (0.2%)	1 (0.2%)	
M stage, n (%)			0.465
M0	171 (46.3%)	173 (46.9%)	
M1	10 (2.7%)	15 (4.1%)	
Gender, n (%)			0.892
Female	139 (27.1%)	137 (26.7%)	
Male	117 (22.8%)	120 (23.4%)	
Race, n (%)			0.559
Asian	2 (0.4%)	5 (1.1%)	
Black or African American	26 (5.8%)	26 (5.8%)	
White	197 (44.2%)	190 (42.6%)	
Age, n (%)			0.104
≤65	109 (22.1%)	129 (26.1%)	
>65	137 (27.7%)	119 (24.1%)	
Smoker, n (%)			0.915
No	38 (7.6%)	36 (7.2%)	
Yes	212 (42.5%)	213 (42.7%)	

**Table 5:** GO analysis of the co-expressed genes of CDK1 and CXCL8.

MCODE	GO	Description	Log10(*p*)
MCODE_1	R-HSA-416476	G alpha (q) signaling events	−17.2
MCODE_1	R-HSA-500792	GPCR ligand binding	−14.5
MCODE_1	R-HSA-388396	GPCR downstream signaling	−13.4
MCODE_2	R-HSA-977225	Amyloid fiber formation	−10.9
MCODE_2	R-HSA-73728	RNA polymerase I promoter opening	−9.2
MCODE_2	R-HSA-5334118	DNA methylation	−9.2
MCODE_3	GO:0045109	Intermediate filament organization	−13.3
MCODE_3	GO:0031424	Keratinization	−12.9
MCODE_3	GO:0045104	Intermediate filament cytoskeleton organization	−12.8
MCODE_4	R-HSA-6809371	Formation of the cornified envelope	−11.9
MCODE_4	R-HSA-6805567	Keratinization	−10.8
MCODE_4	GO:0031424	keratinization	−6.7
MCODE_5	R-HSA-9660821	ADORA2B-mediated anti-inflammatory cytokines production	−9.4
MCODE_5	R-HSA-418555	G alpha (s) signaling events	−9.2
MCODE_5	R-HSA-9664433	Leishmania parasite growth and survival	−9

## Data Availability

Not applicable.

## Supplementary Materials

The following are available online at https://www.mdpi.com/article/10.3390/cells11172688/s1, Table S1: The sample ID associated with the study in the TCGA database; Table S2: Basic information of patients used to verify the expression levels of CDK1 and LINC00261. Table S3: Basic information of patients used to measure IL levels.
